# Exploratory Analysis of Treading Water Coordination and the Influence of Task and Environmental Constraints

**DOI:** 10.3389/fpsyg.2019.02579

**Published:** 2019-12-05

**Authors:** Chris Button, Luka Brouwer, Christophe Schnitzler, Harjo J. de Poel

**Affiliations:** ^1^School of Physical Education, Sport and Exercise Science, University of Otago, Dunedin, New Zealand; ^2^Center for Human Movement Sciences, University Medical Center Groningen (UMCG), University of Groningen, Groningen, Netherlands; ^3^Equipe d’accueil en Sciences Sociales (E3S, EA n°1342), Faculté des Sciences du Sport, Strasbourg University, Strasbourg, France

**Keywords:** aquatic skills, coordination, drowning, life-saving, stability

## Abstract

The radical embodied cognition approach to behavior requires emphasis upon how humans adapt their motor skills in response to changes in constraint. The aim of this exploratory study was to identify how the typical coordination patterns used to tread water were influenced by constraints representative of open water environments. Twenty-three participants were measured while treading water (TW) in a swimming flume in four conditions: (1) in still water, wearing a bathing suit (baseline); (2) wearing typical outdoor clothing (clothed); (3) with an additional cognitive task imposed (dual task); and (4) against a changing current (flow). Mixed methods kinematic analysis revealed four different TW coordination patterns were used across the conditions. The four TW patterns used represent a hierarchy of expertise in terms of the capacity to generate continuous lift forces, where pattern 1 (the lowest skill level) involved predominantly pushing and kicking limb movements (*N* = 1); pattern 2 was a movement pattern consisting of legs pushing/kicking and arms sculling (*N* = 7); pattern 3 was synchronous sculling of all four limbs (*N* = 6); and pattern 4 was the “eggbeater kick” (the highest skill level), with asynchronous sculling movements of the legs (*N* = 9). The four TW patterns were generally robust to the modified constraints. The higher skilled patterns (i.e., patterns 3 and 4) appeared to be the most stable coordination patterns. These results suggest that learning to perform more complex patterns to tread water might be an asset to survive in life-threatening situations.

## Introduction

Drowning is recognized as a significant problem globally (WHO, 2014). Rising sea levels and unpredictable weather patterns due to climate change endanger the lives of many people all over the world (Patz and Kovats, 2002). Each drowning case is multifactorial in terms of contributing factors (i.e., preceding activity, experience, environment, task, etc.) (Croft and Button, 2015). Such factors can be thought of in terms of influential constraints that both limit and enable the emergence of behaviors – an individual’s “aquatic readiness” (Langendorfer and Bruya, 1995). In that respect, treading water (TW) is a foundational movement skill for humans when submerged in water, particularly when external buoyancy aids are not available. TW involves maintaining a stable head position above the water surface by limb movements. The capacity to tread water allows people to monitor themselves and their environment and to make an informed decision about subsequent behaviors, while being able to maintain steady breathing (Golden and Tipton, 2002). While several studies have considered the mechanics of TW among skilled sports people (e.g., Sanders, 1999; Homma and Homma, 2005), less is known about how individuals of various skill levels tread water across different environmental conditions and task demands.

Schnitzler et al. (2014a, 2015) identified four main TW patterns used by individuals in still water. The four patterns were categorized within a typology from theoretically less efficient to more efficient based on the nature of forces created (lift or drag) and the type of interlimb coordination (synchronous or asynchronous) used. In order to generate sufficient buoyancy to keep one’s head above the water surface, drag and lift force are the two kinds of forces predominantly generated (Toussaint and Truijens, 2005). Lift is created perpendicular to the direction of the body’s movement, whereas drag acts in the opposite direction to the body’s movement. Lift forces are predominantly generated through sculling (or sweeping) movements of the hands and feet. To illustrate, sculling is a “propeller” kind of movement with the hands and feet when moving the limbs outward and inward, in which the contralateral limb pairs (i.e., inter-arm and inter-leg) can act in conjoint synchrony or alternation. Furthermore, downward drag can be created with pushing and/or kicking movements. This is however less efficient than sculling, as for instance pushing downward with the arms to drive one’s body upward implies subsequent recovery movements, i.e., the arms need to go upward again; the latter creates (partly) the opposite effect, namely drag in an unwanted direction (Schnitzler et al., 2014a). Therefore, TW patterns that rely upon drag force production are seen as less efficient than those that generate lift forces using sculling movements.

In their classification scheme, Schnitzler et al. (2015) showed how common TW patterns can be ranked according to their posited efficiency. Four main patterns could be identified, of which examples are depicted in the video resources associated with this article (see Supplementary Videos). One of the patterns (dubbed as pattern 1) implies pushing/kicking movements consisting of up and down arm movements and anterior-posterior leg movements, which can be synchronous or asynchronous (i.e., inter-leg/inter-arm coinciding movement or alternation, respectively). This technique appeared to be adopted mainly by less proficient treaders (e.g., not by experienced water polo players). Pattern 2 is a more efficient TW solution than pattern 1 (Schnitzler et al., 2014a) and it is characterized by lateral sculling movements (i.e., generating lift) of either the upper or lower limbs. A more advanced solution involves sculling of both the arms and legs (pattern 3). This pattern is relatively effective for generating lift, but due to the (synchronous) breaststroke kick with the legs when sculling, the buoyancy force produced is (partly) discontinuous. Similar to rowing in a boat, such discontinuity of force generation involves significant power losses, which can however be regained by applying asynchronous movement patterns (e.g., de Brouwer et al., 2013). Continuous generation of lift force can be achieved by using the so-called “eggbeater kick” (pattern 4). Here, the arms make sculling movements near the water surface, while the left and right legs alternate their sculling movements, forming an “alternating breaststroke kick” pattern (i.e., asynchronous inter-leg coordination). Hence, pattern 4 is most effective at generating continuous lift force to support the body and keep the head above the water surface with little up-down oscillations (Sanders, 1999; Homma and Homma, 2005). While this typology of Schnitzler et al. (2014a, 2015) was created through qualitative analysis of people TW while wearing their swimsuits in the (warm) still water of a swimming pool, most drownings do not occur in static water (i.e., swimming pools) but instead in dynamic, open water environments like oceans and rivers (WHO, 2014; Croft and Button, 2015). These environmental constraints might influence the adaptive capacities of a potential victim. The primary aim of the current study was to explore the influence of lab-controlled environmental and task constraints on the stability of TW mechanics.

Taking a radical embodied cognitive science perspective, movement patterns self-organize depending on the environment and the current state of the movement system (Chemero, 2013; Favela, 2014). According to Marsh et al. (2009), consideration of how environmental context evokes meaningful behavior is important, requiring researchers to focus “not just on the body or environment as creating input for the cognitive system, but examining a body’s actions as an object of study in itself” (p. 1220). The non-linear nature of system organization means that up to a certain range of constraint level, human behavior is maintained, hence stable. Once the constraints are out of that range, sudden switching (or a transition) toward a new stable pattern of behavior can emerge (Kelso, 1995). As such, the stability of a movement pattern resides in how well it can be maintained and, hence, is reflected by when and how transitions to other patterns occur. Such transitions can lead to bifurcations or shifts (Kostrubiec et al., 2012) to a pattern more adapted to the set of constraints, as has been established within basic locomotion like terrestrial gait. For instance, at a certain gait speed (i.e., a task constraint considered as a scalable control parameter), the locomotor pattern (order parameter) shifts from walking into running (e.g., Diedrich and Warren, 1995). Furthermore, hysteresis occurs: the walk-to-run transition occurs at a higher speed than for run-to-walk due to the relative stabilities of the two types of pattern (e.g., Hreljac, 1993; Li, 2000). Similarly, for aquatic human locomotion, water flow is a well-known constraint, which also appears to be sensitive to the skill level of the swimmer (Chollet et al., 2000; Seifert et al., 2010). From a water-safety perspective, it would therefore be important to determine whether movement pattern transitions are dependent upon the proficiency of the participants in water. In other words, do more proficient water treaders have inherently more stable movement patterns that are more resistant to constraint changes than less proficient people? A related question is whether humans switch from treading water to a globally different pattern (i.e., swimming) at certain boundaries of water flow or are such transitions and hysteresis effects dependent upon the stability of the pattern that is adopted? To the best of our knowledge, such questions have yet to be investigated, so a secondary purpose of the current study was to explore whether the stability of TW patterns abides by similar dynamical principles as land-based locomotion.

Recent studies have already examined the effects of water temperature as a control parameter on aquatic survival skills (Button et al., 2015; Schnitzler et al., 2018). These experiments showed that cold environments increased the subjective and objective difficulty of the task and impacted negatively the time it could be sustained. However there was no significant modification of the TW pattern used as a function of the temperature. Other constraints could also act as control parameters in the context of aquatic survival skills. For example, Stallman et al. (2013) highlighted that clothing, water flow, or cognitive activity may also influence this motor adaptation in water. Barwood et al. (2011) examined the influence of wearing (different types of) clothing after immersion. They discovered that when dressed, an initial increase of the buoyancy (probably due to trapped air bubbles between clothing layers) gradually dissipated with time. Hence clothing constrained buoyancy differently as a function of time spent in the water. Regarding cognitive load as a control parameter, studies on land-based locomotion have shown that the primary motor task is (negatively) affected when performing a concurrent cognitive task, for example in walking (Ebersbach and Dimitrijevic, 1995; Doi et al., 2010; Ellmers et al., 2016) and obstacle crossing (Worden et al., 2016). Likewise, performing a cognitive dual task may disrupt TW movement patterns and frequency. The dual-task paradigm is a classic manipulation in cognitive science to explore the proficiency of performance on a primary task *via* the addition of a secondary cognitive task (e.g., Kirchner, 1958; Ellmers et al., 2016).

The aim of this exploratory study was to identify how the typical coordination patterns used to tread water were influenced by constraints representative of open water environments. We postulate that four different TW patterns will be identified (based on the taxonomy of Schnitzler et al., 2015). Given that TW is an important, potentially life-saving skill, we propose that the four coordination patterns may be inherently stable (i.e., resistant to external perturbation), albeit to different degrees depending on the mechanical efficiency of the preferred pattern. Hence, one might presume that more stable TW patterns are robust to perturbations and result in fewer transitions to different coordination modes than unstable patterns. Based on indications from land-based locomotion (Diedrich and Warren, 1995) one might hypothesize that there is a transition from one to another TW pattern (or even to swimming) when the water flow increases or decreases. Furthermore, when gradually increasing the current, we speculate that the transition will occur at a higher current than when the water flow speed is decreasing.

## Materials and Methods

### Participants

Twenty-three participants (18–55 years of age, *F* = 14, *M* = 9) were recruited *via* advertisements placed on a university-based website and at local aquatic sports clubs. Participants signed informed consent prior to testing and were only included if they reported themselves as sufficiently competent to tread water without support for at least 5 min. Given this inclusion criterion, the TW expertise of the participants potentially ranged from competent to highly skilled (subsequently verified through qualitative analysis of movement patterns). They also completed a brief health and fitness screening questionnaire (i.e., Physical Activity Readiness Questionnaire). Exclusion criteria included: standing height of over 1.85 m (6′1′′) due to the maximal depth of the flume, self-reported learning difficulties, or existing health conditions (e.g., injuries, severe asthma) that may put the participant at risk during testing.

### Equipment

**Figure 1:**
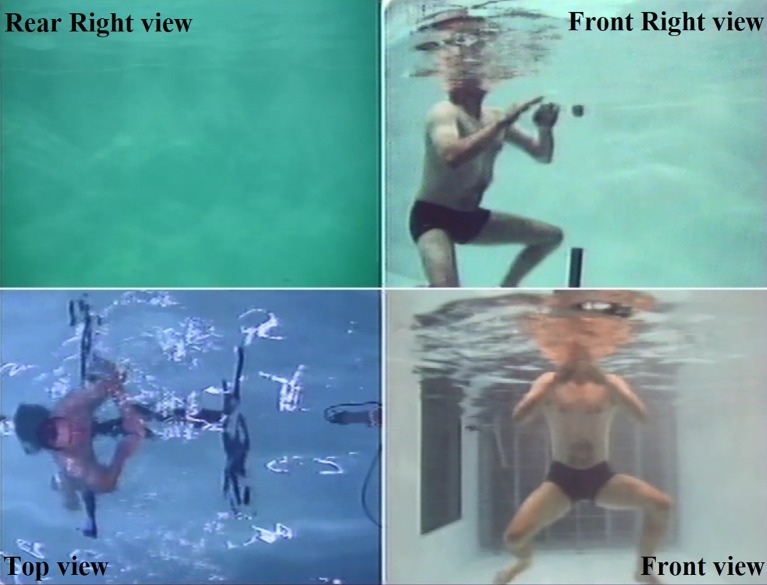
The four synchronized camera views. The front view (bottom right image) was primarily used to determine TW pattern and movement frequency. The rear right view (top left image) was useful in instances where participants drifted from the set position and when they began swimming. These images were captured in the baseline condition. Written permission and informed consent were obtained from the depicted individual for the publication of the image.

Testing occurred in a swimming flume (StreamliNZ, Dunedin, New Zealand). The flume is an aquatic water channel in which the current can be manipulated from still (no flow) to 3.5 m/s. The flume depth was 2 m and a swimming area of 6 m × 2 m was available. During all conditions, the participants were recorded with four video cameras configured to cover the whole swimming area: one from the front of the swim channel, one from above, and two from the right side (see Figure 1). The front camera was the principal camera for analysis, the other cameras were used as back-up in case of equipment malfunction or to help confirm pattern classification. The water temperature was consistently set at 27°C to ensure the data were not influenced by temperature differences (Button et al., 2015; Schnitzler et al., 2018).

The study included four conditions: baseline (BA), clothed (CL), dual task (DT), and a water flow (WF) condition (see Table 1). The BA condition was conducted first, the order of the other conditions was randomized to limit order effects. During the CL condition, participants were asked to wear their own jeans, socks, trainers, t-shirt (short sleeve), and jumper (long sleeve) without hoodie or long zip. The standardization of personal clothing was an attempt to limit differences in clothing affecting buoyancy forces as shown by Barwood et al. (2011). During the DT condition, the participant had to perform an N-back memory task (Kirchner, 1958), which involved recalling whether a presented stimulus was the same as presented N trials ago (a 2-back task was used in the present study). The N-back task is a continuous task typically used to assess working memory capacity. The sequence of stimuli was presented on a TV screen in front of the participant at the edge of the flume at eye height. The verbal response of the participant was recorded by the investigator by clicking a button (if the answer was “yes”) or not clicking a button (if the participant remained silent).

**Table 1:** Descriptions of the experimental conditions.

Condition	Task description
Baseline (BA)	Tread water for 180 s in still water in typical swimwear
Clothed (CL)	Tread water for 180 s in still water while wearing casual clothes (i.e., shoes/trainers, jeans, t-shirt, and a jumper) over typical swimwear
Dual task (DT)	Tread water for 180 s in still water in typical swimwear while performing the “visual 2-Back” task for 120 s, starting 30 s after the start of treading water. Participants were asked to prioritize treading water (primary task) over the performance of the 2-back task (secondary task)
Water flow (WF)	Tread water for 30 s in still water in typical swimwear. Beginning from still (no flow), the current was increased every 30 s (0-0.4-0.6-0.8-1 m/s) for 150 s and then decreased with the same increments (to 0 m/s) for a further 150 s.

During the WF condition, the current of the water within the flume was modified in small increments, from still to a moving, unidirectional flow. The participants wore a belt around their chest, which was secured to a bar at the front of the flume by an elastic rope (at water level) to maintain their overall position within the flume channel. They were positioned to face the oncoming water flow approximately 4 m in front of a safety net. Previous research had shown that recreational swimmers can comfortably maintain their position by swimming in the flume at 1 m/s (Button et al., 2015). Therefore, the current was incrementally increased up to approximately 1 m/s and then decreased till 0 m/s, in steps of 0.2 m/s every 30 s, except for the first step (and last step), which was 0.4 m/s due to practical limitations of the flume turbines.

### Procedure

This study was carried out in accordance with the recommendations of World Medical Association’s Declaration of Helsinki. The protocol was approved by the University of Otago’s Human Ethics Committee (Reference: 16/158). The experimental design is based on a multiple repeated measures model (baseline condition and three perturbation conditions). Before starting the experimental conditions, a number of personal and anthropometric variables were collected (i.e., height, weight, buoyancy, gender, age, and ethnicity data) (Table 2).

**Table 2:** Mean participant characteristics (±SD).

	Age (years)	Height (m)	Weight (kg)	Buoyancy (N)
Men (*N* = 9)	37.6 (10.9)*	1.75 (0.06)*	75.46 (13.46)	9.30 (0.18)*
Women (*N* = 14)	28.4 (7.1)*	1.69 (0.06)*	73.62 (17.04)	9.48 (0.16)*
Overall (*N* = 23)	32.0 (9.7)	1.72 (0.06)	74.34 (15.44)	9.41 (0.19)

* *Significant difference between men and women (p < 0.05)*.

Given the potential for individual buoyancy to influence pattern stability in the current study, aquatic body weight was determined for all participants. Static buoyancy was determined in a drop tank. A plastic chair was attached *via* a strain gauge (Futek LCM300 250lb., Futek Advanced Sensor Technology Inc., USA, sample frequency: 100 Hz) to a mechanized winch that lowered the chair into the tank. Participants were asked to sit in the chair above water level with a 5-kg weight belt worn around the waist to stabilize the participant’s position on the chair when submerged. The chair was then winched in the water until the participant’s chin was just above water level. Once the participant and chair were steady, measurements of buoyancy (corrected for weight of participant and weight belt) were recorded for up to 20 s, while participants held their breath (to limit movement). To obtain reliable data, this procedure was undertaken three times with rests permitted between attempts.

All testing conditions (see Table 1) occurred on one occasion, approximately 60 min in duration. In each condition, participants were asked to tread water and keep their head position as stable as possible above the water surface (without any further instructions regarding how to move). In the WF condition, participants wore a chest strap and were advised to adopt whatever movement pattern felt most comfortable to maintain their stable head position. Additional to this basic task requirement, the nature of the experimental conditions was explained to the participants in advance.

### Data Analyses

The raw data supporting the conclusions of this manuscript will be made available by the authors, without undue reservation, to any qualified researcher.

#### Qualitative Analysis

Two analysts (authors CB and LB) were trained to perform qualitative analysis of TW according to the method developed by Schnitzler et al. (2014b). This method is based on the observation of different patterns of interlimb movement. According to these authors, less skilled patterns are based on the use of drag force and synchronous movements to stay afloat, whereas more skilled patterns are characterized by the use of lift force and asynchronous movements to generate small but frequent force momentum to ensure stability (Schnitzler et al., 2014b). This can be observed as upside-down (pushing) movements for the least skilled participants, whereas more skilled participants exhibit lateral movement (sculling) from the legs and the hand. Both analysts independently identified each of the four TW coordination patterns from the random sample of participants chosen. Each analyst performed qualitative analysis on a portion of the data independently and the inter-rater reliability was 100% (*к* = 1.0, *p* = 0.00). This inter-rater reliability was calculated from a random sample (17%) of the data.

The predominant coordination patterns adopted in the BA, CL, and DT conditions were identified from video footage. The “*preferred*” pattern was defined as that used for the longest duration throughout each condition. Furthermore, when an additional pattern was observed for three or more movement consecutive cycles within the same condition, it was classified as a “*pattern change*.” The number of pattern changes within a condition were counted and used as an indication of the stability of the main pattern. Within the WF condition, the identification of patterns was done once for every water flow velocity increment (i.e., nine times in total). Since swimming of some form was necessary at least at the highest current (1 m/s), any identifiable swimming patterns were recorded, along with the TW coordination patterns. Each velocity increment lasted only 30 s (to minimize fatigue); therefore, no pattern changes were counted within each WF bin. However, by identifying the pattern used in each step, it could be seen at which current the transition to another pattern was made.

On visual inspection, it became clear that some participants almost never changed their movement pattern, regardless of experimental condition. To check the stability of movement patterns, we could therefore *a posteriori* divide the participant pool into two subgroups (“changers” and “non-changers”) based on the amount of changes made within conditions and if they used a different preferred pattern in the CL, DT, or (the first step of) the WF condition than used in the BA condition. If participants changed their movement pattern at least three or more times from the BA condition, they were allocated to the “changer” subgroup. All other participants were categorized into the “non-changer” subgroup.

#### Quantitative Analysis

The buoyancy data were filtered (Butterworth 4^th^ order, cut-off frequency 0.5 Hz) and divided by the participant’s weight to calculate standardized values. For the BA, CL, and DT conditions, the movement frequency of the arms and legs during treading water was determined. This frequency analysis was done by visual inspection using the video-recorded data and the average time of nine movement cycles. Three movement cycles were taken just after 30 s, three cycles just after 60 s, and three cycles just after 90 s. For the WF condition, the changing current predominantly determined limb movement frequency; hence, the frequency analysis was not performed in this condition.

#### Statistical Analysis

The TW patterns were initially categorized based on the preferred pattern adopted in the BA condition (i.e., patterns 1–4 according to the typology of Schnitzler et al., 2014b). Arm and leg frequency in the BA condition was compared between the four types of patterns with a MANOVA. The total number of pattern changes in each condition were also counted (i.e., the number of times there was a shift to an extra pattern and/or back to the main pattern) and participants were then allocated into two subgroups (“changers” and “non-changers”). The pattern distribution used in the two categories was compared graphically with violin plots and also with Kruskal-Wallis tests.

We were also interested in exploring whether a hysteresis effect exists for aquatic locomotion. In the WF condition, the currents at which transitions between treading water and swimming occurred were recorded and compared. A paired sample *t*-test was run to contrast the current at which the transition from TW to swimming (increasing) occurred in relation to the transition from swimming to TW (decreasing). All statistical tests were carried out using SPSS Statistics 23.0. A significance level of *α* = 0.05 was adopted for all tests. Partial eta squared ($\eta_{p}^{2}$) or Cohen’s *d* was reported as an estimate of effect size (Richardson, 2011) and Bonferroni-corrected *t*-tests were used as *post hoc* analysis where applicable.

## Results

Eighteen out of 23 participants (78%) performed all experimental conditions successfully, five were not able to and/or chose not to finish all WF stages. All dependent variables were checked for the assumptions of parametric tests and only weight did not meet these assumptions. Further analysis identified a single outlier for weight which was not removed from further analyses since the buoyancy variable was normally distributed. Mean data in Table 2 show that males and females were different in terms of age [*F*(1,21) = 6.00, *p* < 0.03, $\eta_{p}^{2}$ = 0.22]; height [*F*(1,21) = 4.61, *p* < 0.05, $\eta_{p}^{2}$ = 0.18]; and buoyancy [*F*(1,21) = 6.57, *p* < 0.02, $\eta_{p}^{2}$ = 0.24]. However, there were no significant sex differences in terms of the patterns adopted over conditions, and the frequency of arms and legs in the BA condition. It was therefore assumed that sex did not have a significant influence upon the coordination patterns adopted and was not included in the remaining analysis.

### Identification of Coordination Patterns in the Baseline Condition

The distribution (N) of participants who performed each coordination pattern in the baseline (BA) condition is depicted in the leftmost column of Table 3. As only one participant performed pattern 1, this participant’s data were not included in further statistical analysis due to uneven group sizes. As can be seen from Table 3, movement frequency of the legs differed between patterns [*F*(2,19) = 9.89, *p* < 0.01, $\eta_{p}^{2}$ = 0.51]. *Post hoc* tests confirmed that leg frequency was higher in pattern 4 compared to patterns 2 and 3 (Bonferroni: *p* = 0.002/*p* = 0.01, respectively). There is also a tendency for higher arm frequency in pattern 4 compared to patterns 2 and 3 with an effect size considered as small (Cohen, 1988), but this trend failed to reach significance [*F*(2,19) = 3.25, *p* = 0.061, $\eta_{p}^{2}$ = 0.26].

**Table 3:** Average movement frequency of arms and legs (±SD) and the total number of pattern changes in the baseline condition (BA).

	Arms (Hz)	Legs (Hz)	Pattern changes
Overall (*N* = 23)	0.79 (0.22)	0.80 (0.25)	28 (*N*_c_ = 5)
Pattern 1 (*N* = 1)	0.71 (0.00)	0.83 (0.00)	7 (*N*_c_ = 1)
Pattern 2 (*N* = 7)	0.74 (0.26)	0.63 (0.13)*	9 (*N*_c_ = 2)
Pattern 3 (*N* = 6)	0.66 (0.23)	0.68 (0.24)*	12 (*N*_c_ = 2)
Pattern 4 (*N* = 9)	0.92 (0.12)	1.01 (0.18)*	0 (*N*_c_ = 0)

*N_c_ depicts the number of participants that showed within-condition pattern changes*.

* *Significant difference between pattern 4 and either 2 or 3 in leg frequency (p < 0.05; Bonferroni: p = 0.002/0.010, respectively)*.

### Influence of Constraints Upon Coordination Patterns

**Figure 2:**
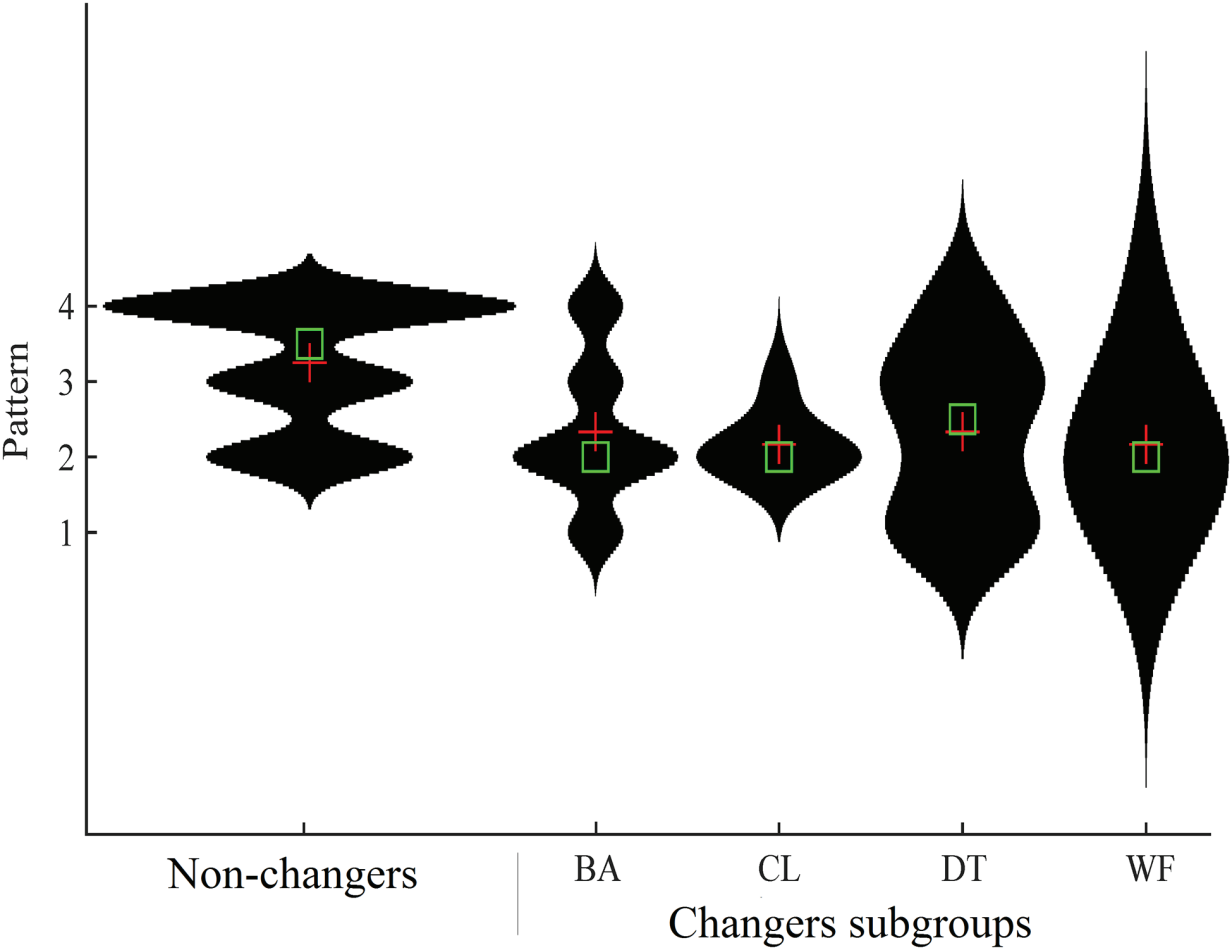
Violin plots of TW pattern distributions for the Non-Changers and Changers subgroups. The green boxes denote the median and the red crosses denote the mean average. The wider the shape, the more frequently the TW patterns were expressed. The longer the shape, the larger the interquartile distribution.

In the BA condition, five participants made changes to their TW patterns (Table 3). On the basis of whether participants made pattern changes from their preferred pattern in the CL and DT conditions, two more participants were identified as “changers” (*N* = 7, 30%). Kruskal-Wallis tests identified differences in coordination patterns between the subgroups in the CL condition [*χ*^2^(1) = 5.85, *p* < 0.05, $\eta_{p}^{2}$ = 0.23] and the WF condition [*χ*^2^(1) = 4.52, *p* < 0.05, $\eta_{p}^{2}$ = 0.17]. The seven changers tended to use less efficient TW patterns (i.e., mostly pattern 2) compared to the non-changers subgroup (mostly patterns 3 or 4) (Figure 2).

Three out of 23 participants (13%) changed TW pattern within the CL condition compared to their preferred BA pattern. Interestingly, there were fewer overall pattern changes in the CL condition as compared to the BA condition (18 vs. 28 in total). Furthermore, only two participants (7%) changed their TW pattern in the DT condition compared to the BA condition. There were only eight changes within the DT condition, compared to 28 within the BA condition. Although not reported here for brevity, analysis of the N-back cognitive task revealed minimal decrements in the performance of the secondary task (maintained at between 100 and 90% for all but one participant).

**Figure 3:**
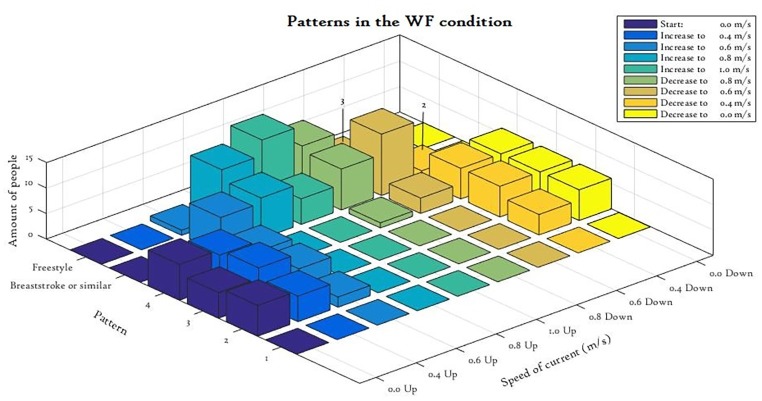
3D bar chart depicting shifts to other TW patterns, breaststroke or freestyle in the WF condition.

In the WF condition, all 18 participants (who completed this condition) started using one of the four patterns listed in Table 3. When the water started to flow, only four participants (22%) changed their TW pattern before starting to swim. No clear order of swimming techniques (breaststroke and freestyle) was used at the higher currents, since some participants started with breaststroke and then freestyle, while others only used breaststroke or freestyle. In Figure 3, the overall course of movement pattern transitions is shown. As expected, all participants changed their movement from TW to swimming when the current increased and back to TW when the current decreased. Overall, there were 58 transitions between patterns in this condition with each participant making between two and four changes.

The transition current was different between the increasing and decreasing flow [*T*(17) = 8.79, *p* < 0.001, Cohen’s *d* = 2.1]. The transition tended to occur at a higher current (mean = 0.72 ± 0.10 m/s) in the increasing current condition than when the current was decreasing (mean = 0.40 ± 0.18 m/s). Participants who changed from either pattern 2 or pattern 3 to swimming transitioned at the same or higher current than from swimming back to TW. However, for individuals performing pattern 4, this group effect was not the same since they were already treading water at 0.8 m/s with decreasing current, while there were no such patterns present at 0.8 m/s with increasing current (indicative that the hysteresis effect is inversed for pattern 4).

## Discussion

This exploratory study considered how TW patterns were adapted to altered task and environmental constraints. In general, the results suggest that people use robust TW movement patterns that do not easily change to other patterns when constraints change. Overall four TW patterns were identified (Table 3), in line with past studies (Schnitzler et al., 2014a, 2015). The stability within conditions seemed highest among the nine individuals (39%) performing pattern 4. Furthermore, the eight participants (35%) using the least efficient patterns (patterns 1 and 2) displayed more transitions within and between conditions (Figure 2). It therefore seems that participants using pattern 4 were the least vulnerable to disruptions and that this “eggbeater kick” would be the more stable coordination mode that is resistant to changes in constraints. The radical embodied cognition approach to behavior emphasizes how humans learn to move adaptively as constraints change (e.g., Chemero, 2013) and this study provides another excellent illustration of this phenomenon.

In the current research, we explored the effects of a (continuous) cognitive demand on TW performance by using a dual task. However, the dual task did not disrupt the TW patterns (since changes were less frequent in the DT than the BA condition) and the performance of the dual task was not harmed either (>90% correct on average). Previous research on land-based locomotion had indicated that the performance of the primary task (i.e., walking) can be disrupted by the addition of a secondary cognitive task (Ellmers et al., 2016). One interpretation of this discrepancy is that primary task activities that are visually guided (like walking) are more vulnerable to dual-task disruption than those that do not rely heavily on continuous visual regulation (like TW). Another possible explanation is that participants were able to freely switch attention between the dual tasks without significantly disrupting performance of the coordination pattern used in the primary task (see Verhaeghen and Basak, 2005). Since pattern changes in the DT condition were less frequent than in BA, the DT condition in this research might serve more like a real-life “baseline,” since individuals typically do something else while TW (e.g., talking or planning their next behavior). The BA condition of this research might have been too monotonous, which invited individuals to explore (see Newell, 1986) and try out other ways to tread water and therefore more shifts in the BA condition than in DT resulted. Furthermore, in real-life drowning situations, typical dual task scenarios would likely be much more demanding (i.e., planning a survival strategy, weighing up risks against benefits, etc.) and anxiety levels would be higher than during the DT condition of this study.

It was notable that changes in coordination do occur when the current of the water alters. Rather than transitioning from TW to swimming due to spatial restrictions of the flume, we believe that participants change because it becomes a more streamline (efficient) position to comfortably adopt in the moving water. Movement patterns of low stability levels are more vulnerable to transitions as has been shown for example in human hand movements (e.g., Kelso, 1984; Haken et al., 1985) and locomotion on land (e.g., Hreljac, 1993; Diedrich and Warren, 1995; Li, 2000). Individuals performing the eggbeater pattern (pattern 4) were able to maintain treading water in faster flowing conditions compared to individuals performing the other TW patterns (Figure 3). However, note that these pattern transitions due to water flow were mainly changes between treading water and swimming, not (often) between the four different TW patterns.

Not only did individuals using pattern 4 maintain it at higher speeds in the WF condition, they also made no changes within the BA condition and were more often categorized in the non-changers group. We interpret these findings as indicative of greater relative stability in pattern 4 compared to the three other patterns. Nevertheless, the movement frequency of the legs was higher compared to the other patterns (Table 3), so pattern 4 might be more physically demanding. As the asynchronous sculling of legs putatively generates smaller lift forces (albeit continuously) in contrast to the synchronous pattern 3, a quicker cycling action (i.e., a higher movement frequency) is required to maintain the head position above water. Consequently, if in a survival situation an individual needed to tread water for extended periods of time, the more stable pattern may not necessarily be the most efficient pattern to adopt. It is also likely that the stability of this pattern might be related to specific experience, since pattern 4 is often used by water polo players and synchronized swimmers (Sanders, 1999; Homma and Homma, 2005). It will be important for future research to compare the relative benefit to be gained from using the different TW patterns particularly in terms of energy efficiency and past experience. Additionally, an important future consideration will be the extent to which vertical and horizontal transfer exists between skills such as treading water and associated activities like swimming.

Our results also show for the first time that a hysteresis effect may exist between TW and swimming, which is mediated by TW expertise. In more detail, the transition from TW to swimming tended to occur at a higher current than when switching back to TW in patterns 2 and 3 (see Figure 3), whereas the transition from TW to swimming in pattern 4 occurred at a lower current than when switching back to TW. One interpretation of this indicative finding is that pattern 4 possesses more inherent stability than the other three patterns and is more resistant to the external perturbation of water flow. Further research is needed to formally model and confirm the indicative hysteresis effect more thoroughly than we have been able to in this exploratory study.

The design of the study and limited instructions provided helped to characterize the intrinsic dynamics of the participant’s behavior in an ecological situation. Importantly, participants were not told how to tread water but simply to maintain a stable position in the water. Had we instructed participants to resist transitions between patterns as long as possible, then different behaviors might have resulted, but that was not the main focus of the study. This analysis of emergent behavior is typical of previous dynamic systems research and extends land-based treadmill studies to aquatic locomotion (e.g., Kelso, 1981; Kelso, 1984; Diedrich and Warren, 1995). Still the question remains: do we need to change patterns to be able to cope with the different aquatic circumstances regarding dynamic, open water environments? A few participants mentioned that they started to swim in the WF condition just because the flume eventually had an “end” (safety net) to avoid, which may not be the case when immersed in a river or sea. Therefore if in open water, these participants mentioned they would just go with the flow and keep themselves afloat. Resisting a current might not be the most effective strategy to survive (e.g., when caught in a tidal rip), but keeping the head above the water and not panicking does seem important, whether you “go with the flow” or not.

### Limitations

In this study, we tried to recreate typical constraints that might affect the capacity for people to tread water in open water situations. However, closely simulating all features of open water situations in a flume was not possible. In open water, there is no need to stay at the same place in the current most of the time, but due to material conditions of the testing environment the participants had to avoid moving toward the end and sides of the flume. While the spatial restrictions imposed may have admittedly influenced behavior (as they undoubtedly do in treadmill locomotion), the control procedures employed were necessary for logistic and safety reasons. It is also possible that fatigue may have influenced whether participants made transitions between patterns particularly among less skilled participants. As fatigue was not a focus of this investigation (albeit an important topic worthy of future consideration), the procedure was designed to limit the amount of time exercising in each condition to no more than 5 min and with ample opportunity to rest between conditions. Furthermore, anxiety undoubtedly plays an influential role in most survival situations, but for ethical reasons fear could not be induced within these controlled laboratory-based settings. Lastly, buoyancy forces will vary among the population for example due to different weather conditions and clothing worn (Barwood et al., 2011). For comparison between participants, a standard set of clothing was imposed, but that limits generalization to all immersion situations in which clothing is varied. Despite such limitations due to the testing conditions, it is important to know the potential disruptions typical constraints can have on TW. This knowledge will help in further research about the prevention of drowning.

### Practical Implications

This study suggests that different TW patterns may be expected from the general population and that such movement patterns are fairly robust to different circumstances. Some patterns are more effective at generating lift force and resisting the influence of altered constraints. We showed that the “eggbeater kick” was the most stable pattern although it is not necessarily the easiest (most familiar) pattern to produce. The leg kick lateral sculling movements and asynchronous coordination thereof may mean that this pattern requires considerable practice and instruction to perform effectively. When designing a representative training environment, water safety instructors should try to enrich practice with different sets of constraints, i.e., by asking trainees to tread water in different directions at different speeds and with and without clothing. As cognitive function does not seem to be hampered by treading pattern, it seems advisable to create scenarios that promote problem solving and decision-making while practicing TW. Finally, it is important to note that a stable movement pattern could be life-preserving in a threatening situation.

## Data Availability Statement

The datasets generated for this study are available on request to the corresponding author.

## Ethics Statement

The studies involving human participants were reviewed and approved by Human Ethics Committee, University of Otago. The participants provided their written informed consent to participate in this study.

## Author Contributions

CB conceived the experiment, co-supervised LB’s Master’s project, and wrote the final draft of the article for submission. LB conducted the data collection and data analysis and lead wrote the first draft of the article. CS provided advice on qualitative analysis and data interpretation, as well as editing the final draft. HP instigated the project by organizing LB’s project in New Zealand, provided advice on experimental design, and also edited the final draft.

### Conflict of Interest

The authors declare that the research was conducted in the absence of any commercial or financial relationships that could be construed as a potential conflict of interest.

## Supplementary Material

Supplementary Video 1 Exemplary video of treading water pattern 1 according to the classification scheme of Schnitzler et al. (2015).

Supplementary Video 2 Exemplary video of treading water pattern 2 according to the classification scheme of Schnitzler et al. (2015).

Supplementary Video 3 Exemplary video of treading water pattern 3 according to the classification scheme of Schnitzler et al. (2015).

Supplementary Video 4 Exemplary video of treading water pattern 4 according to the classification scheme of Schnitzler et al. (2015).
